# Point-of-Care Diagnostic for *Trichomonas vaginalis*, the Most Prevalent, Non-Viral Sexually Transmitted Infection

**DOI:** 10.3390/pathogens12010077

**Published:** 2023-01-03

**Authors:** John F. Alderete, Hermes Chan

**Affiliations:** 1 School of Molecular Biosciences, Washington State University, Pullman, WA 99164, USA; 2 MedMira, Suite 1, 155 Chain Lake Drive, Halifax, NS B3S 1B3, Canada

**Keywords:** α-actinin, control vertical line (CVL), diagnostic target, MedMira, monoclonal antibody (MAb), point-of-care (POC), serodiagnosis, sexually transmitted infections (STIs), *Trichomonas vaginalis*, Rapid Vertical Flow (RVF^®^) Test Technology

## Abstract

A point-of-care (POC) diagnostic is needed for both women and men to establish universal screening and surveillance for the number one, non-viral sexually transmitted infection (STI) caused by *Trichomonas vaginalis*. We developed a POC diagnostic for this STI using the MedMira Rapid Vertical Flow (RVF^®^) Technology test cartridge with a membrane that includes a Vertical procedural/reagent control line (referred to as CVL) and spotted with 1 µg of a 72.4-kDa truncated version of α-actinin called ACT::SOE3. This protein is a specific diagnostic target for antibody in sera of individuals with trichomoniasis. Serum antibody to ACT::SOE3 is a positive reaction with the test spot. Specificity of ACT::SOE3 was revealed with monoclonal antibodies (MAbs) generated to ACT::SOE3. Addition of negative control serum with MAb 67B reactive to ACT::SOE3 shows detection of both ACT::SOE3 and the CVL. Only positive sera of individuals had antibody reactive with ACT::SOE3 and detected the presence of the spot and the CVL. Negative control sera were unreactive with ACT::SOE3 and only showed the presence of the CVL. Importantly, to show proof-of-principle for POC application, ACT::SOE3 was detected with the positive patient sera spiked with whole blood. Finally, packaged cartridges stored with desiccant packs at 37 °C for one year gave identical results with the positive and negative human sera. Our results show the validity of this new POC serodiagnostic for this STI.

## 1. Introduction

World Health Organization reports have emphasized the significance and impact of the estimated curable 376 million STIs on human health [1,2,3,4]. *Trichomonas vaginalis*, the causative agent for trichomoniasis, is the number one, non-viral STI with an estimated incidence of 156 million yearly cases worldwide followed by chlamydia (127 million), gonorrhoea (87 million), and syphilis (6 million) [1,3,5]. As of 2016, the prevalence of these STIs was high with *T. vaginalis* numbers among women exceeding those of men in all country income categories [4,6]. For individuals with trichomoniasis, incidence rates were highest in the regions of Africa, the Americas, Eastern Mediterranean, and Western Pacific.

In the United States in 2000 there were ~19 million incident cases of STIs, and 9.1 million were 15 to 24 years of age [7]. The extent of the prevalence and incidence numbers of *T. vaginalis* STIs among the age groups 15 to 59 years are high and indicates that this STI is both underreported and neglected [8,9,10,11]. This is evident also in the studies that show the high numbers of undiagnosed, persistent infections, especially within certain racial/ethnic groups, such as among African Americans in certain settings [10,11,12,13,14]. In a study it was found that 50% of pregnant women had undiagnosed, persistent infections [13].

Most *T. vaginalis* STIs are asymptomatic, which contributes to chronic subclinical and adverse health outcomes for individuals. This STI causes maternal and perinatal morbidity and mortality, ectopic pregnancy, pelvic inflammatory disease, adverse birth outcomes, and congenital infections [2,15,16]. *Trichomonas vaginalis* infections enhance the acquisition of HIV, which is disproportionately higher among African Americans [2,17,18,19,20,21]. Recent literature continues to suggest a relation between *T. vaginalis* and prostate cancer [22,23,24,25,26,27,28]. These facts reinforce the need for enhanced routine and universal screening to prevent the adverse morbidity by this STI in our country and worldwide.

The POC OSOM™ *T. vaginalis* rapid test (Sekisui Diagnostic, San Diego, CA, USA) is an immunochromatographic, lateral flow diagnostic that is in use in the United States and in other countries [29,30,31]. This test is only for women and is invasive, requiring a vaginal swab and remains underutilized. Therefore, there is a need for a POC diagnostic for this STI for individuals infected with *T. vaginalis* and, importantly, that fulfills the WHO ASSURED criteria [32]. This paper presents a 72.4-kDa truncated version of the highly immunogenic *T. vaginalis* α-actinin protein [33] called ACT::SOE3 [34,35] as a target for a new serodiagnostic for trichomoniasis. ACT::SOE3 is an ideal target for an α-actinin blood antibody-based POC test for several reasons. For example, individuals with trichomoniasis make serum IgG antibodies to the natural 106.2-kDa immunogenic α-actinin [33]. α-Actinin is not found among other microorganisms and has no identity with the human homolog [34,35]. Further, sera of individuals with trichomoniasis are unreactive by various assays with the human homolog α-actinin homolog protein [34,35]. Finally, no serum antibody is found to *T. vaginalis* α-actinin among uninfected women and men [34,35]. This fact makes ACT::SOE3 a target for a blood antibody-based POC test [34,35]. Here, we present a POC diagnostic for this STI using the MedMira Rapid Vertical Flow cartridge with a membrane that includes a Vertical procedural/reagent control line (referred to as control vertical line or CVL) and a spot that is readily visible within a 5 min reaction with α-actinin antibody present in serum or whole blood specimen.

## 2. Materials and Methods

### 2.1. MedMira Rapid Vertical Flow (RVF^®^) Core Technology

**Figure 1:**
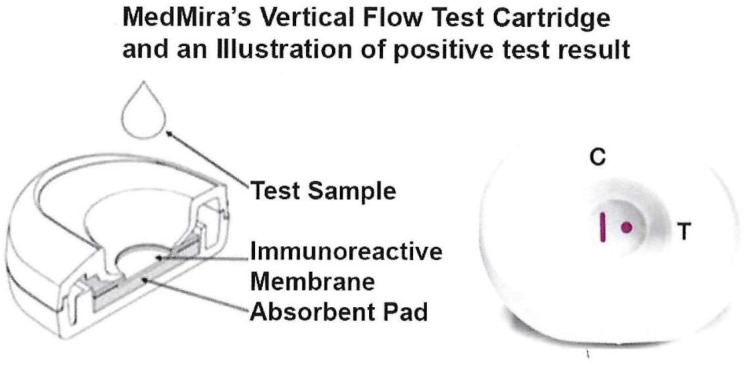
Illustration of the test cartridge consisting of two plastic cases within which is the nitrocellulose membrane overlaid on top of the absorbent pad (**left side**). The top and bottom halves snap together to hold the nitrocellulose membrane that sits on top of the pad. The **right side** shows a cartridge with a positive test result illustrated by the antibody detection of the spot with immobilized analyte (labeled T). The CVL (labeled C) captures antibody in serum that is also detected by the InstantGold cap with gold-conjugated Proteins A and L without interfering with the antigen-binding site. This indicates the end of the functional platform test and shows a positive result using patient serum or whole blood. Visualization of the CVL but absence of a spot is a negative result with no antibody in serum or blood to the analyte.

The RVF Core Technology test has been reported before [36,37,38,39]. The ACT::SOE3 recombinant protein at a concentration of 1 mg/mL is formulated in PBS, pH 7.5, containing 0.1% Tweet 20, 0.6% SDS, 10 mM EDTA and 0.01% of sodium azide. It is then mechanically immunoprinted as a dot of this buffered cocktail on a nitrocellulose membrane (MDI, India) that has been printed with a proprietary formulated Protein A control vertical line (CVL) (Figure 1). Both capture antigen cocktail and the CVL are immunoprinted on the membrane using a Biodot-Biojet Quanti3000 Dispenser (EN0311).

The InstantGold Cap consists of a plastic housing that holds a specific low-protein affinity filter (MDI India) onto which a proprietary formulation of detection reagent has been immobilized. The detection reagent is comprised of Proteins A and L Colloidal Gold Conjugate (PALCG) that are manufactured internally by MedMira. These are commonly used reagents in diagnostic tests and are comprised of two components, which serve equally important but different roles. Proteins A and L functions to provide specificity to the reagent as it binds specifically to immunoglobulins IgG and IgM. The conjugated colloidal gold allows the binding to be visualized through the red color.

The Universal Buffer solution, which is the third and final key component, is a multifunctional buffer solution that is used to complete the testing steps. This buffer is a proprietary formulation of ionic and non-ionic detergents in Tris-buffered saline with preservative. The buffer serves four major functions, as follows: (1) to lyse red blood cells in whole blood specimens, (2) to optimize the interactions between capture antigens on the test membrane and antibodies present in the specimen, (3) to reduce non-specific binding to the membrane that produces clearer test backgrounds, and (4) to reconstitute the dried colorimetric detection agent contained in the InstantGold Cap and facilitate its flow through the test membrane where the assay reaction occurs.

### 2.2. Whole Blood for Testing for Antibodies to ACT::SOE3

The test requires whole blood for screening women and men for seropositivity to *T. vaginalis* α-actinin. A drop of blood must be mixed with buffer 1 prior to adding to the platform and followed by placement of the cap and addition of buffer 2. The kit includes a sterile blood lancet with a double-edged blade and pointed end. The lancet makes a puncture like a fingerstick to obtain a small blood specimen that is added to the vial 1 buffer. The lancet is then disposed appropriately.

### 2.3. α-Actinin and ACT::SOE3 and Monoclonal Antibodies (MAbs)

The *T. vaginalis* α-actinin (106.2-kDa) and descriptions and methods for obtaining ACT::SOE3 (72.4-kDa) used for this diagnostic have been described [34,35]. ACT::SOE3 was purified from lysates prepared from pellets of recombinant *E. coli* using Ni^2+^-NTA superflow affinity column chromatography (Qiagen Inc., Valencia, CA, USA), as before. Purification of the protein was assessed using sodium dodecylsulfate polyacrylamide gel electrophoresis [34,35,40,41]. At the Core Facility of the College of Veterinary Medicine mice were immunized with purified ACT::SOE3 to generate MAbs using established protocols and as previously described [35]. This was approved by the Washington State University Biosafety Committee of the Institutional Review Board (number 01058). All animals were treated humanely (Institutional Animal Care and Use Committee number 6317).

### 2.4. MAbs and α-Actinin Antibody in Serum for ACT::SOE3 Detection

Five microliters of each of the hybridoma supernatants of the new MAbs 15B, 67B, and 74B to ACT::SOE3 were mixed with buffer 1 and added to the cartridge. The InstantGold cap was then placed on the cartridge followed by addition of buffer 2. When buffer was not visible, the cap was removed, and detection of the red spot showed a positive reaction with MAb. Irrelevant MAbs do not detect the ACT::SOE3. All MAbs were of the IgG_1_ isotype.

The use of positive sera from women (n = 25) and men (n = 25) for detection of the natural α-actinin and truncated ACT::SOE3 have been detailed earlier [34,35,40,41,42,43,44]. Sera were chosen based on ELISAs on microtiter plates coated with α-actinin and were done in quadruplicate, as before [34,35,40,41,42,43,44]. Sera of women (n = 25) and men (n = 25) negative for ACT::SOE3 were used to show absence of false positives, as before [33,34,40,41,42,43,44]. Fifty microliters of individual positive or negative woman and man serum were mixed with buffer 1 and added to the cartridge. After penetration of buffer 1 into the cartridge, buffer 2 was added to the InstantGold caps. Positive serum detection of the ACT::SOE3 spot was evident by reaction with gold-conjugated Proteins A and L to give a red spot on the diagnostic cartridge. All negative women and men sera were unreactive with ACT::SOE3.

### 2.5. Origin of Sera

Positive and negative men sera to α-actinin were obtained from Washington University at St. Louis, MO [42,44] and were from a repository of the Health Professionals Follow-up Study, which began in 1986. Men sera were also from the Harvard T.H. Chan School of Public Health obtained for The Physician’s Health Study that begin in 1982 for examining the prevention of cardiovascular disease and cancer [43]. These studies were used to also examine for relationships between infectious diseases and prostate cancer. Men were not initially examined for infection by *T. vaginalis*. Approval was obtained from each universities Institutional Review Boards.

Sera and blood of individuals infected with *T. vaginalis* and uninfected controls were provided by Ob/Gyn clinician-scientists of the School of Medicine at the University of Texas Health Science Center (UTHSCSA) at San Antonio. Confirmation of infection by *T. vaginalis* was by microscopy and growth of parasites using the InPouch^®^ (Biomed Diagnostics, White City, OR, USA). These sera were used in numerous prior studies [34,35,40,41,42,43,44]. As above, the Ob/Gyn clinician scientists of the UTHSCSA obtained approval from the university Institutional Review Board. Informed consent forms from all subjects were also obtained.

### 2.6. Reproducibility

The Rapid Vertical Flow Tests (RVF^®^) for ACT::SOE3 were performed on three separate occasions under identical conditions and all assays gave identical positive and negative reactions.

## 3. Results

### 3.1. RVF^®^ Test Technology Cartridge and Single Analyte Detection Platform

Figure 1 (left side) shows the test cartridge consisting of two plastic casings that snap together to securely hold the reactive nitrocellulose membrane overlaid on top of the absorbent pad. The assembled cartridge (right side) illustrates a control vertical line (CVL, labeled C) and the Test Zone spot (labeled T). Both the CVL and spot are shown in red to represent the positive reaction with serum or blood containing antibody. The CVL contains an optimized amount of Protein A that is immobilized on the nitrocellulose membrane to allow non-specific capture of Ig antibodies as a control. InstantGold cap contains colloidal gold-conjugated Proteins A and L that enables the visualization of the Ig antibodies formed on the reactive membrane.

The advantage of this platform in serum or whole blood antibody detection is that excess materials flow through the membrane away from the reaction zone to permit visualization of bound components. This represents a POC test that uses simple reagents, eliminates false results, and is easy to read. Further, the test requires no dilution of serum or blood, no equipment, and no trained personnel. Importantly, the test can be performed within five minutes. The test is stable at room temperature for more than one year.

### 3.2. Rapid Vertical Flow (RVF^®^) Test Package and Materials

**Figure 2:**
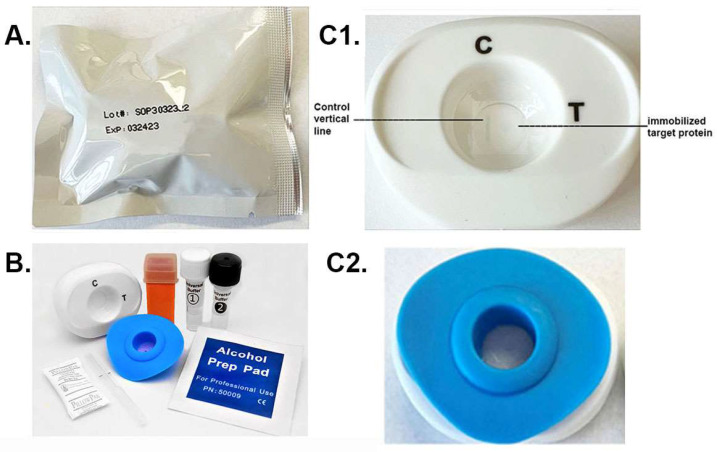
The MedMira RVF test package and components. (**A**) displays the package containing the diagnostic materials. (**B**) shows the cartridge (white) and InstantGold caps (blue) containing gold-conjugated Proteins A and L for color development. The sterile lance (orange) is provided for obtaining a drop of blood. Buffer 1 is mixed with serum or whole blood prior to addition to the cartridge shown in (**C1**). (**C2**) shows the InstantGold cap placed on the cartridge for addition of buffer 2.

The package with the patented MedMira RVF^®^ Technology toolkit is shown in Figure 2A. Within the package are the materials needed to perform the diagnostic using serum or whole blood specimen. These include the white test cartridge and the blue InstantGold cap (Figure 2B). Also included is the lance (orange) for obtaining the drop of blood specimen and the buffers 1 and 2. The enlarged picture of the cartridge (Figure 2C1) shows the CVL, which binds Ig in serum or blood, and the spot where the target protein is immobilized is next to the CVL. The InstantGold cap placed on the cartridge is shown in part Figure 2C2. These packages with desiccant are stable at room temperature for up to one year.

### 3.3. Step-Wise Approach

**Figure 3:**
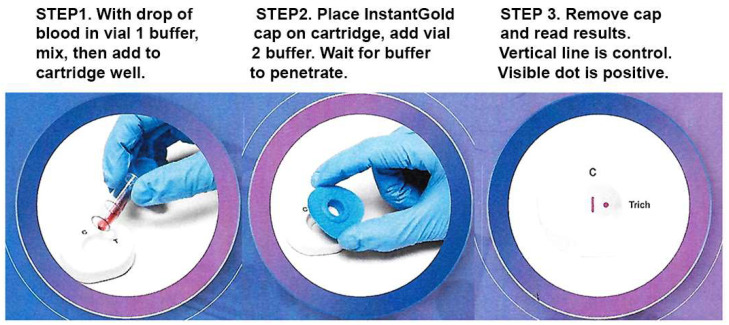
The three steps of the test are illustrated in sequence. The CVL contains immobilized Protein A to capture antibodies in blood and serves as a procedural and reagent control. The presence of antibody to ACT::SOE3 permits visualization of the red spot as a positive reaction.

Figure 3 shows the three steps for performing the diagnostic. A drop of blood is mixed with vial 1 buffer to lyse cells (Step 1). The mixture is added to the cartridge well. After buffer penetrates the membrane, the InstantGold cap is placed on the cartridge well followed by addition of vial 2 buffer for release of the gold-conjugated Proteins A and L (Step 2). The cap is then removed for visualization of the protein spot and the CVL (Step 3).

### 3.4. Specific MAb Detection of ACT::SOE3

We tested for the binding of MAbs 67B, 74B and 15B that were generated to ACT::SOE3 for detection of the protein immobilized on the nitrocellulose and compared with the control, irrelevant MAbs ALD30 and L64 of the same IgG_1_ isotype. One microgram of ACT::SOE3 in PBS was spotted onto the cartridge nitrocellulose membrane. For this experiment 5 µL of hybridoma supernatant was mixed with buffer 1 and added to the cartridge followed by placement of InstantGold caps and addition of buffer 2. The MAbs 67B, 74B and 15B detected ACT::SOE3, as expected [34,35]. The irrelevant MAbs ALD30 and L64 did not detect the protein. The MAb ALD30A is to an epitope of the trichomonad glyceralde-3-phosphate dehydrogenase [40,41,45], and MAb L64 reacts with an epitope of a small-sized cytoplasmic protein of the parasite [46].

**Figure 4:**
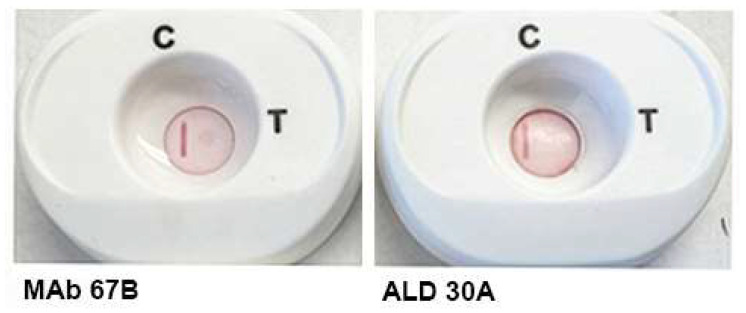
Visualization of the CVL and spot after addition to buffer 1 of 50 µL of a negative serum and 5 µL of positive MAb 67B (**left side**). Buffer 1 with serum and negative MAb ALD30A (**right side**) shows the CVL but no reaction with ACT::SOE3.

Next we repeated the experiment using positive MAb 67B and negative MAb ALD30A but with 50 µL of negative serum added to buffer 1 and processed as above. As expected and seen in Figure 4, only MAb 67B again detected ACT::SOE3, but importantly, the CVL was now evident for both platforms.

### 3.5. Cartridges with Positive Reactions to Different Sera

**Figure 5:**
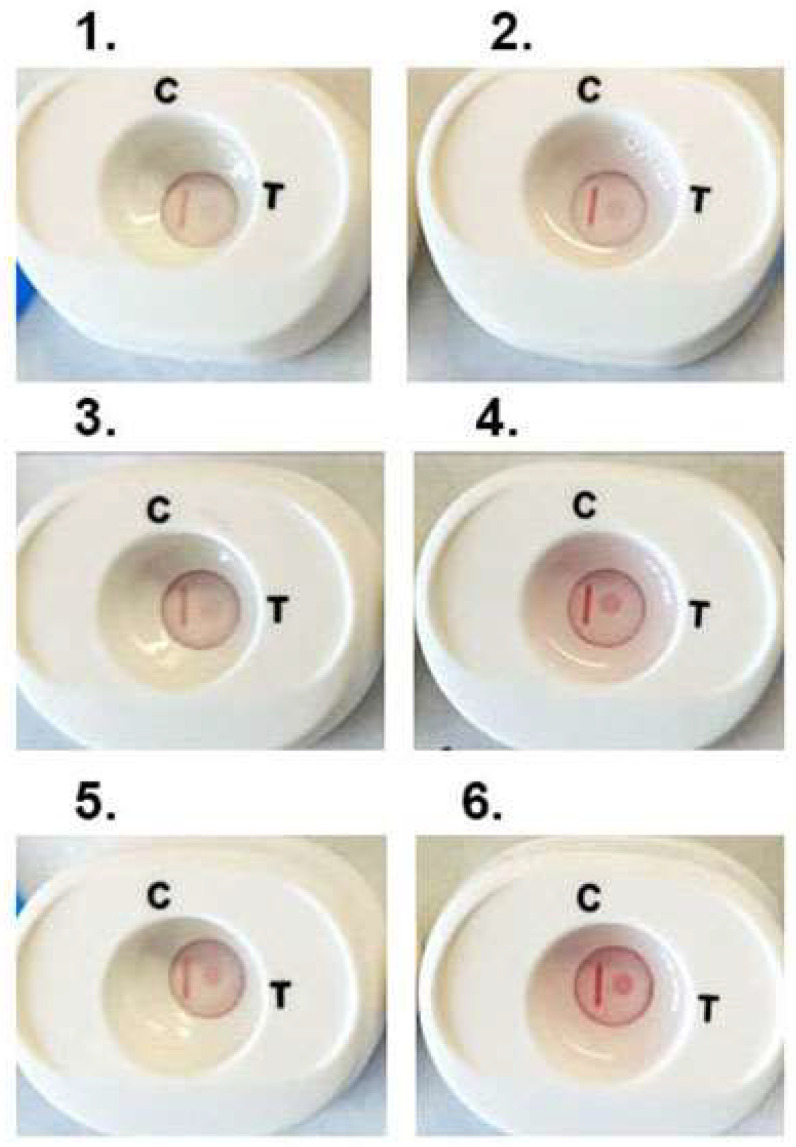
Representative experiment showing the detection of ACT::SOE3 with 3 each woman (**left side**, numbers (**1**,**3**,**5**)) and man (**right side**, numbers (**2**,**4**,**6**)) positive serum. Negative women and men sera as previously defined are unreactive with ACT::SOE3 [34,35,40,41,42,43,44].

We then performed numerous tests on platforms with individual positive woman and man serum, as before [34,35,40,41,42,43,44]. Fifty microliters of respective serum samples were added to buffer 1 and mixed and cartridges processed. Figure 5 is a representative experiment with three individual woman (1, 3 and 5) and man (2, 4 and 6) positive serum that detect ACT::SOE3 and show the CVL. All six cartridges show the detection of the ACT::SOE3 at different intensities, suggesting different amounts of antibody to the protein.

### 3.6. Use of Cartridges with Blood-Spiked Positive and Negative Sera

As the platform is designed to be a serodiagnostic test using a drop of blood, it was important to perform assays using four pooled positive and negative women and men sera. Each pooled sera were derived from equal volumes of 4 different serum samples. Fifty microliters of each of the pooled sera were mixed with a drop of human whole blood and combined with buffer 1 to lyse blood cells before addition to the cartridge. Results were read after placement of the InstantGold caps on the cartridges and addition of buffer 2.

**Figure 6:**
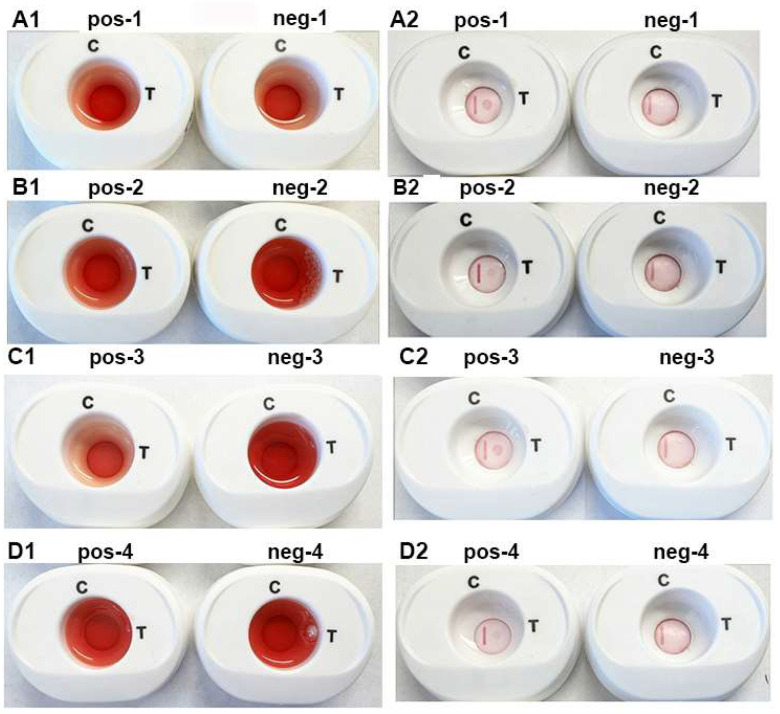
Representative experiment to illustrate corresponding reactions of individual positive (pos-1 through pos-4) and negative (neg-1 through neg-4) pooled women (**A1**,**A2**,**B1**,**B2**) and men (**C1**,**C2**,**D1**,**D2**) sera in the presence of a drop of blood. Platforms (**A1**–**D1**) has buffer 1 mixed with 50 µL of pooled sera and a drop of blood and added to the cartridges. After the blood penetrates the membrane, the reaction is developed by placement of the InstantGold caps followed by addition of buffer 2. After the cap is removed, the results are presented in (**A2**–**D2**) for both the positive and negative sera. Only the positive sera detected the ACT::SOE3. The CVL was visible for all cartridges.

Figure 6 is a representative experiment using the individual pooled positive (pos-1 through pos-4) and negative (neg-1 through neg-4) women (parts A1,A2,B1,B2) and men (parts C1,C2,D1,D2) sera. The serum with a drop of blood is presented in panels (A1–D1). After penetration of buffer 1, buffer 2 was added to the InstantGold caps. Panels (A2–D2) (pos-1 through pos-4) show detection of ACT::SOE3 with positive sera. No signal was evident for the negative sera (neg-1 through neg-4). In all platforms the CVL was readily visible.

## 4. Discussion

This paper shows ACT::SOE3, the truncated version of the highly immunogenic α-actinin protein [33], as a new POC serodiagnostic target for trichomoniasis. Epitope mapping identified the 13 epitopes detected by sera of women and 5 epitopes detected by sera of men, which are a subset of the 13 [34]. The epitopes have no amino acid sequence identity with other proteins of microorganisms and the human homolog in databanks. Importantly, individuals infected with *T. vaginalis*, but not uninfected individuals, make serum antibody to the trichomonas α-actinin protein. Also noteworthy is that the α-actinin gene and protein amino acid sequence of *T. vaginalis* are invariant regardless of origin of the isolates and length of time during batch cultivation [34,35]. Finally, and importantly, the human oral trichomonad, *T. tenax*, and the intestinal *Pentatrichomonas hominus* were not detected by immunoblots of total proteins using as probes the MAbs to ACT::SOE3 [35], and antiserum to *T. tenax* from mice immunized with either live organisms or extracts of organisms was unreactive by ELISA to purified ACT::SOE3 (unpublished data), reaffirming the utility of this diagnostic protein target for trichomoniasis. Therefore, all individuals infected with *T. vaginalis* will have serum antibody to the epitopes within the ACT::SOE3 diagnostic target.

The extent of the prevalence and incidence of this *T. vaginalis* STI in all age groups is high and indicates that this STI is both underreported and neglected [8,9,10,11,12,13,14]. Women 45 years and older were diagnosed and treated for *T. vaginalis*, and one-third of the women had persistent infection diagnosed within one year of treatment [12]. Fifty percent of pregnant women have increased risk of undiagnosed, persistent infections concomitant with numerous adverse health outcomes [13,15,16]. An STI Survey Program in Baltimore, MD showed the prevalence of trichomoniasis was 7.5% overall and 16.1% for African American women, most of whom were asymptomatic [10]. These and other studies show the high numbers of undiagnosed, persistent infections among African Americans in certain settings [9,10,12,13,14]. This is significant because of the consequences to the reproductive health of women [12,16,17,18,19,20,21]. These facts reinforce the view that what is needed is an increased awareness for greater emphasis of routine and universal screening to prevent the adverse morbidity by this STI in our country and worldwide.

An important issue that needs to be addressed is the temporal nature and duration of the serum anti-*T. vaginalis* antibody responses among individuals after infection and after diagnosis and cure. There is the possibility of long-lasting serum antibody, which may not be indicative of a current infection. It has been shown that there is short-lived serum and vaginal antibody to *T. vaginalis* proteinases after treatment of patients [47,48]. Additionally, there appears to be a decline in the titer of antibodies after treatment of infected individuals, although antibody was still detected from one to three months [49,50]. In this study an enzyme immunoassay (EIA) with whole trichomonads was used for antibody screening, which may explain the long-lasting antibody. For example, it has been established that serum of individuals infected with *T. vaginalis* and immunized animals possesses antibodies to many cytoplasmic and surface proteins of *T. vaginalis* [47,48,51,52,53,54]. Thus, it is conceivable that the immune responses would produce long-lasting antibodies, albeit not high-titered, to some proteins that, using a whole cell-EIA, would be detected weeks after cure. Therefore, a POC test with a specific protein, like ACT::SOE3, would be a better marker regarding the kinetics of antibody responses following *T. vaginalis* STIs and after treatment and cure.

Importantly, serological tests as proposed here for this POC serodiagnostic have been used historically for the diagnosis of infectious diseases and continue to have notable advantages [55,56]. For example, the direct detection of *T. vaginalis*, such as by microscopy, is problematic, and the detection of antibody may help to diagnose an acute infection. Serological surveys using a POC serodiagnostic give an immediate epidemiological measure of the STI among a population [55,56]. While a positive OSOM™ test may be indicative of active infection [29,30,31], this α-actinin serodiagnostic test may be used for screening of large, at-risk populations [8,9,10,13,14]. This is especially important because of the asymptomatic, persistent nature of trichomoniasis and where testing is limited only for symptomatic cases. Antibody to α-actinin, which is invariant protein unique to this organism, is evidence of a response to infection. In addition, serological screening for this STI may identify more infections than reported cases alone. Equally noteworthy is that data from such a serological survey may inform public health agencies to develop strategies for interventions to decrease and/or eliminate the reproductive health morbidity attributed to this STI [12,15,16,17,18,19,20,21]. Non-invasive serological screening for *T. vaginalis* is directly applicable for direct use by health workers that delivers immediate results and may provide a learning and collaborative opportunity between the health professionals and infected individuals. Finally, a simple, easy-to-use serum test for serological screening eliminates challenges experienced among laboratories, such as methodological gaps that lead to quality of testing issues when using different assays and the absence of testing reagents in resource poor countries [57,58].

This new vertical flow test for serodiagnosis of trichomoniasis fulfills the WHO ASSURED criteria [32]. It is a low-cost, rapid test that is user-friendly, transportable, equipment-free, and sensitive and specific. Importantly this test can be adopted for use with multiple perfect serodiagnostic targets to *T. vaginalis*. For example, the novel chimeric epitope chain protein called AEG::SOE2 was recently invented [40], and this chimeric protein is comprised of immunogenic epitopes of fructose-1,6-bisphosphate aldolase (A), α-enolase (E), and glyceraldehyde-30phosphate dehydrogenase (G). These epitopes of AEG::SOE2 are unique to *T. vaginalis* [40]. Thus, AEG::SOE2 may be added onto the membrane as a separate dot next to ACT::SOE3 (Figure 1). In this scenario, having two diagnostic target proteins with epitopes from four different proteins without identity to other proteins would enhance the validity of diagnosis for this STI. Furthermore, such a test with multiple targets could simultaneously provide more accurate, additional data regarding the kinetics of antibody responses. Equally noteworthy is that this diagnostic can be configured into a multiplex test for detection of multiple STIs on the same cartridge. Finally, it is plausible that this new POC test may become a new WHO universal standard for trichomoniasis diagnosis so that *T. vaginalis* will no longer be considered a neglected STI [11].

## 5. Conclusions

The burden of the prevalence and incidence of *T. vaginalis* in our country and worldwide makes evident the need for a POC test that fulfills the WHO ASSURED criteria. This is especially highlighted by the chronic, subclinical adverse health outcomes for individuals with trichomoniasis. Equally significant are the findings of undiagnosed, persistent *T. vaginalis* infections and health disparities among subpopulations. This work presents a serodiagnostic test for this STI using a form of the α-actinin protein, ACT::SOE3, which has been shown experimentally to be a target for detection of serum antibody. It is evident that this test can be used in all geographic settings. This test may be an important tool for health care providers and individuals infected with *T. vaginalis* that will hopefully enhance the routine and universal screening of all age groups and of at-risk populations, which hopefully will enhance the reproductive health of women and men.

## 6. Patents

JFA:

No. 9,910,042, “Strings of Epitopes Useful in Diagnosing and Eliciting Immune Responses to Sexually Transmitted Infections.” Inventor J.F. Alderete, Issued to Washington State University 6 March 2018.

No. 10,386,369, “Strings of Epitopes Useful in Diagnosing and Eliciting Immune Responses to Sexually Transmitted Infections.” Inventor J.F. Alderete, Issued to Washington State University 20 August 2019.

No. 10,677,798. Strings” of Epitopes Useful in Diagnosing and Eliciting Immune Responses to Sexually Transmitted Infections.” Inventor J.F. Alderete, Issued to Washington State University 9 June 2020.

HC: (MedMira)

No. 2,493,616. Rapid Diagnostic Device, Assay and Multifunctional Buffer. Canada.

No. ZL02819646.5. Rapid Diagnostic Device and Assay. China.

No. EP1417489. Rapid Diagnostic Device and Assay. Europe (Great Britain, Denmark, Spain, France, Ireland)

No. 7,531,362, Rapid Diagnostic Device, Assay and Multifunctional Buffer. United States.

No. 8,287,817 (InstantGold cap). Rapid Diagnostic Device, Assay and Multifunctional Buffer. United States

No. 8,586,375 (alternate direct labels). Rapid Diagnostic Device, Assay and Multifunctional Buffer. United States.

No. 11,353,450, Rapid Vertical Flow Technology-surface-enhanced Raman spectroscopy. United States.

## Data Availability

No new data beyond what is presented in this paper were created or analyzed in this study. Data sharing is not applicable to this article.
